# Chinese learning self-efficacy (CLSE) among international students in China: group differences across sociodemographic and academic variables

**DOI:** 10.3389/fpsyg.2026.1761904

**Published:** 2026-10-07

**Authors:** Ling Shen, Yiying Wu

**Affiliations:** Chinese Language and Culture College of Huaqiao University, Xiamen, China

**Keywords:** affective attitudes, Chinese learning self-efficacy, cognitive abilities, international students, learning strategies, self-efficacy, social support

## Abstract

Academic self-efficacy significantly impacts the academic performance of international students in China. This research explores the current status and influencing factors of Chinese learning self-efficacy (CLSE) among this population. Based on Bandura’s self-efficacy theory and existing mature scales, this study constructs an analytical framework comprising four dimensions: cognitive ability, affective attitude, learning strategy, and social support. The study then uses SPSS 26.0 to analyze data collected from 427 valid questionnaires. The findings of the questionnaire indicate that the CLSE of international students is generally at a high level. Among the four dimensions, affective attitude self-efficacy is the lowest, while social support self-efficacy is the highest. International students’ CLSE shows significant differences across gender, age, Chinese ethnicity status, educational level, foreign language proficiency, and study duration.In addition to individual influencing factors, including changes in learning motivation, confidence gaps caused by insufficient social support, mismatches between teaching content and actual cultural needs, and intercultural anxiety, all of the above are factors that affect the self-efficacy of international students. This study puts forward targeted recommendations for individuals, schools, and society to improve the CLSE of international students.

## 1 Introduction

Bandura (1997), an American psychologist, systematically proposed the concept of self-efficacy and examined its role in behavioral change and adaptation. This concept has been extensively addressed in Bandura’s subsequent publications and research. Bandura asserted that self-efficacy constitutes a critical component of the self-regulatory system for specific activities, directly influencing the associated psychological processes. Bandura (1986) further underscored the significance of cognitive abilities, affective attitudes, learning strategies, and social support in shaping behavior. Zimmerman and Martinez-Pons’s (1990) self-regulation model of self-efficacy is closely aligned with Bandura (1986) framework. Zimmerman’s research illustrates how learners improve self-efficacy through self-regulation. Pajares and Kranzler (1995) classified the concept into three dimensions: cognitive, motivational, and affective, investigating how these dimensions interact to influence students’ self-efficacy and academic performance.

Academic self-efficacy constitutes an extension of Bandura’s concept of self-efficacy. Scholars have undertaken in-depth explorations and established a clear definition for this construct. They have identified multiple driving variables that influence self-efficacy, including but not limited to social support, family background, teacher evaluations, and peer interactions. These factors can affect an individual’s self-efficacy through various mechanisms. The outcome variables encompass academic performance, emotional states, and learning motivation. Research findings from scholars demonstrate a consensus regarding the influence of self-efficacy on these variables (e.g., Schunk, 1983; Pajares and Kranzler, 1995; Zimmerman and Martinez-Pons, 1990, Zimmerman et al., 1992; Pintrich and DeGroot, 1990; Bian, 2003; Chi and Xin, 2006; Jia et al., 2020; Wei, 2004; Li, 2001; Wang et al., 2001; Liu, 2018). Some researchers have focused on the relationship between self-efficacy and other related concepts (e.g., Zhou and Dong, 1994; Ren, 2022; Zheng et al., 2023). Their studies consistently reveal that an individual’s interest and motivation level toward a task are closely linked to their learning self-efficacy. Strong learning motivation can enhance one’s learning self-efficacy and improve academic performance.

Scholars have developed an array of scales and questionnaires for the assessment of individual self-efficacy. The General Self-Efficacy Scale (GSES), co-developed by German psychologist Schwarzer and his colleagues (Schwarzer and Jerusalem, 1995), was initially constructed as a unidimensional measurement instrument. Subsequently, domestic and international scholars have developed a wealth of relevant assessment scales (e.g., Sherer et al., 1982; Bian, 2004; Bandura, 2006; Li, 2023).

Against the backdrop of China’s rapid economic development in recent years, the global popularity of the Chinese language has maintained a continuous growth trend. Data released on the official website of the Ministry of Education of the People’s Republic of China indicates that the number of academic degree-seeking international students in China showed a significant upward trend from 2001 to 2023. The annual enrollment of academic degree-seeking international students was only 32,002 in 2001, and rose to 333,072 in 2019, achieving nearly tenfold growth. Affected by the pandemic, enrollment also saw a slight decline to 273,792 in 2020, and then gradually recovered to 254,019 in 2023. The growth of enrollment reflects the evolution process of China’s academic education for international students from scale expansion to steady development, and also demonstrates the dynamic balance between the carrying capacity of educational resources and the global demand for international education in China.

International students in China primarily engage in studying the Chinese language and acquainting themselves with Chinese culture. The cultivation of self-efficacy in Chinese learning is essential for international students’ academic achievements and their study abroad experiences. This self-efficacy enables students to genuinely believe in their capacity to achieve anticipated goals, thereby contributing to the enhancement of their ultimate learning outcomes. Domestic scholars have predominantly focused their research on self-efficacy within the context of international Chinese education, analyzing relevant factors from diverse perspectives. Despite the variation in research focuses, these studies have substantially encompassed nearly all influencing factors associated with the teaching process. A large body of prior scholarship has verified that learning self-efficacy constitutes a critical constituent of learning outcomes, and exerts a pivotal influence on both the learning process and academic achievement (e.g., Li, 2018; Zhang, 2020; Cui and Hu, 2022; Yuan and Wang, 2022; Xie, 2022).

The self-efficacy of international students is influenced by a confluence of factors, encompassing language proficiency, cultural adaptation, and the learning environment. A comprehensive understanding and enhancement of international students’ self-efficacy in Chinese learning can significantly contribute to their academic success and overall satisfaction with their study abroad experiences. Furthermore, such efforts can furnish valuable references and insights for the reform of Chinese language teaching methodologies and provide substantive support for the learning processes of international students.

Nevertheless, research examining the influence of family factors or prior experiences (such as learning other languages) on the self-efficacy of international students in Chinese language education remains scarce. Furthermore, existing studies predominantly concentrate on local students or international students from Europe and America, often lacking adequate consideration of the unique cultural adaptation variables that impact self-efficacy within the international student population.

This study examines the current status, group differences, and influencing factors of Chinese learning self-efficacy (CLSE) among international students in China. The research is grounded in Bandura’s social cognitive theory and informed by Zimmerman’s self-regulated learning model and Pajares’ foundational work. Three primary methodological approaches were employed: a literature review to synthesize existing theoretical and empirical findings; a questionnaire survey, adapted from Bian Y. F.’s Academic Self-Efficacy Scale and revised for cross-cultural applicability, which was distributed via the Wenjuanxing platform to students at H University; and statistical analysis utilizing SPSS 26.0 for reliability and validity testing, descriptive statistics, difference tests, and regression analysis. Pursuant to the aforementioned research design, this study primarily addresses the following three questions:

What is the general level of CLSE among international students in China?

Are there significant differences in CLSE among international student groups when categorized by gender, age, ethnic Chinese or non-Chinese, educational level, prior experience learning other foreign languages, and duration of Chinese language study?

Which factors significantly predict the CLSE of international students?

## 2 Methods

This study synthesizes the dimensions of self-efficacy proposed by Bandura (1986), Zimmerman and Martinez-Pons (1990), and Pajares and Kranzler (1995), classifying them into four distinct dimensions: cognitive abilities, affective attitudes, learning strategies, and social support. Cognitive abilities refer to an individual’s capacity to employ thinking, memory, reasoning, and problem-solving skills during the learning process. These faculties facilitate the effective processing of information, thereby enhancing learning efficiency and outcomes. Affective attitudes encompass the emotional and motivational states of learners, such as interest, motivation, confidence, and anxiety, which significantly influence their engagement in Chinese language learning. Positive attitudes can heighten motivation and improve effectiveness, whereas negative attitudes may adversely affect learning results. Learning strategies denote the systematic approaches individuals adopt to acquire, organize, comprehend, and apply knowledge, including task planning, time management, and note-taking. Effective strategies aid in structuring learning content, increasing efficiency, and strengthening retention. Social support entails the assistance provided by others—such as teachers and peers—in the learning environment, which may be emotional, informational, or instrumental in nature. A robust support system can bolster learners’ confidence and motivation, assist in overcoming challenges, and thereby foster learning self-efficacy. In summary, perceived self-efficacy fosters more favorable outcomes in Chinese language acquisition by shaping learners’ behavioral choices, motivational investment, cognitive processes, and emotional reactions.

### 2.1 Basic content of the questionnaire

To ensure accurate comprehension of the survey items by international students, the questionnaire was administered in a bilingual format. It comprises two primary sections: the respondents’ demographic information (18 items) and the CLSE scale (41 items).

The questionnaire primarily encompasses information on gender, age, educational level, year of enrollment, ethnic Chinese status, duration of Chinese language study, duration of extracurricular Chinese learning, engagement in part-time or internship work related to Chinese, scholarship status, current HSK level, study of languages other than one’s native language and Chinese, reasons for learning Chinese, the extent of family members’ understanding of Chinese and China, and the presence of a Chinese learning partner. Among these, the extent of family members’ understanding of Chinese is a multiple-choice question; current HSK level, educational level and grade, and year of enrollment are fill-in-the-blank questions; and the remaining items are single-choice questions.

Given the widespread recognition of the academic self-efficacy scale developed by Bian (2003, 2004) within the Chinese academic community, and following multiple rounds of revision, this instrument not only demonstrates high reliability and validity but is also particularly suitable for measuring self-efficacy within the learning domain. Consequently, the CLSE Scale (CLSES) was adapted from Bian’s (2003, 2004) original work. Considering the original scale’s complex structure and the present study’s focus on international students’ Chinese learning self-efficacy, the number of items was reduced from 63 to 41. This adaptation aimed to enhance response rates while preserving the scale’s core dimensions, item integrity, reliability, and validity. Furthermore, the general learning context was explicitly specified as Chinese language learning. Prior to its application, the adapted scale underwent review and was approved by four experts in International Chinese Language Education. Employing a five-point Likert scale to facilitate respondent comprehension, this instrument extracts dimensions including cognitive abilities, affective attitudes, learning strategies, and social support, incorporating characteristics of international students and aligning with the survey’s focus on CLSE To mitigate the risk of participants selecting identical options across different dimensions, the items were presented in a randomized order, with varying numbers of items assigned to each dimension.

Following the basic information section, the scale comprises four dimensions: cognitive abilities (11 items, specifically items 21, 23, 25, 31, 34, 35, 36, 37, 49, 56, and 57), affective attitudes (17 items, specifically items 19, 20, 22, 24, 27, 29, 32, 38, 41, 45, 50, 51, 52, 53, 54, 58, and 59), learning strategies (7 items, specifically items 26, 28, 33, 42, 46, 47, and 55), and social support (6 items, specifically items 30, 39, 40, 43, 44, and 48), resulting in a total of 41 items.

The questionnaire adopts a five-point Likert scale, with response options ranging from strongly agree to strongly disagree. Responses are coded numerically from 1 to 5. Items 22, 35, 36, 38, 50, 51, 52, 58, and 59 are reverse-scored. Consequently, a higher level of CLSE is inversely associated with the total score.

### 2.2 Questionnaire implementation

Employing a convenience sampling method, participants were recruited from H University, a major host institution for international students in China. This study adheres to the principles outlined in the Declaration of Helsinki and received approval from the Institutional Review Board of the University. Data collection was conducted via online questionnaires, administered through the Wenjuanxing platform, which combined demographic items with the CLSE scale. The survey was distributed between April 15 and June 15, 2024, utilizing student WeChat groups and class-specific communication channels. Prior to participation, written informed consent was obtained from all respondents, with assurances provided regarding anonymity and confidentiality. All collected responses underwent manual quality screening. Questionnaires were excluded from analysis if they were incomplete, exhibited uniform response patterns across all items, or provided evidence of random or inattentive responding.

Following data cleaning, 427 valid questionnaires were retained (477 in total), yielding an effective response rate of 89.5%. Given that the scale comprises 41 items, the obtained sample size (approximately 10 times the number of items) satisfied the criteria for conducting factor analysis and tests of group differences. The sample encompassed key demographic variables, including gender, age, educational level, HSK proficiency, and ethnic Chinese background, with subgroup sizes adequate for subsequent between-group comparisons.

### 2.3 The used statistical tests

All statistical analyses were performed using SPSS 26.0 software. Internal consistency was evaluated through Cronbach’s Alpha coefficients. The suitability of the data for factor analysis was examined employing the KMO measure and Bartlett’s Test of Sphericity. Subsequently, exploratory factor analysis was conducted to verify the theoretically hypothesized four-dimensional structure, which encompasses cognitive ability, affective attitude, learning strategies, and social support. Descriptive statistics were calculated to summarize the central tendency and dispersion of both the overall CLSE scores and the scores for each specific dimension. To investigate group differences, one-way analysis of variance (ANOVA) was utilized to compare mean scores across demographic subgroups. Statistically significant findings were further analyzed using LSD post-hoc multiple comparisons to identify the precise sources of variation between groups.

As presented in Tables 1, 2, the overall Cronbach’s *α* coefficient was 0.904, indicating good internal consistency. The KMO measure of sampling adequacy was 0.907, and Bartlett’s test of sphericity was significant (*p* < 0.05), confirming the data’s suitability for factor analysis. Subsequently, only data meeting these quality criteria were subjected to further statistical procedures. For the independent-samples *t*-tests conducted in group comparisons, Levene’s test for equality of variances was performed prior to each analysis to examine the homogeneity of variance assumption. For comparisons where Levene’s test was not significant (*p* > 0.05), the results of the standard *t*-test were reported. For the few comparisons where Levene’s test was significant (*p* < 0.05), Welch’s corrected *t*-test was used for statistical inference. Similarly, for all one-way ANOVA analyses across different demographic subgroups, Levene’s test was conducted to verify the homogeneity of variances assumption across groups. All ANOVA analyses satisfied the assumption of equal variances (all *p* > 0.05). Additionally, visual inspection of histograms and Q-Q plots indicated that the distribution of scores within groups did not show marked departures from normality. The established reliability and validity of the questionnaire thus provide a robust foundation for subsequent analytical procedures.

**Table 1:** Overall reliability analysis of the questionnaire.

Reliability statistics		
Cronbach alpha	Cloning of the alpha of Cronbach based on the standardized items	Items
0.904	0.905	41

**Table 2:** KMO and Bartlett’s test for overall validity analysis of the questionnaire.

KMO sample adequacy measure	Approximate chi-square	0.907 4,425.364
Bartlett’s test of sphericity	Degrees of freedom (df)	820
	Sig.	0

### 2.4 Basic information of survey participants

Demographic analysis of this survey reveals that 427 international students are predominantly female (62.5%) and undergraduates aged 18–22 (64.6%). Among them, 64.3% are undergraduates while 22.7% are postgraduates, preparatory students account for about 13%, indicating a relatively young demographic. Non-ethnic Chinese students account for 68.1%, with 53.2% lacking HSK certification and only 6.1% reaching HSK Level 6 proficiency, reflecting generally weak Chinese language foundations.

Regarding investment in Chinese language learning, 59.3% of students have studied Chinese for 1–3 years, with 29.5% falling within the 2–3 year range. Extracurricular self-study time is predominantly concentrated at 1–2 h (38.9%), whereas 17.8% dedicate more than 2 hours. Engagement in part-time work or internships related to Chinese is reported by only 13.6% of students. Within the surveyed cohort, 40% have family members with no knowledge of Chinese, 34.7% lack a basic understanding of China, and merely 15.7% have family members who have previously resided in China. Furthermore, 82% of students do not have fixed Chinese learning partners, suggesting a comparatively limited social support network. Learning motivation exhibits a pragmatic orientation, with “completing studies” (57.8%) and “job seeking” (56.2%) constituting the primary purposes for learning Chinese, while “interest in Chinese language or culture” accounts for 42.9%. Scholarships are received by 73.3% of students. Among scholarship recipients, 34.4% indicate that their scholarships are linked to academic credits, and 44.7% report that scholarship eligibility is directly tied to Chinese language academic performance, reflecting a concurrent presence of academic pressure and incentive mechanisms.

Furthermore, 45% of students possessed prior foreign language learning experience before commencing their Chinese studies, whereas 55% had no such background. In terms of extracurricular self-study duration, students dedicating 30–60 min (29.2%) and under 30 min (14.1%) collectively constituted 43.3% of respondents, suggesting a limited capacity for autonomous learning among a portion of the student cohort. Concerning familial knowledge of Chinese, 25.1% of families reported the ability to comprehend but not speak the language, 19% possessed basic communication skills, and 15.9% of family members were familiar only with isolated vocabulary without understanding semantic content. Regarding the comprehension of Chinese culture, 32.3% of family members, despite not residing in China, expressed an appreciation for it, and 17.3% had traveled to the country. Pertaining to the duration of language learning, 18.3% of students had studied Chinese for 6 months to 1 year, 30% for one to 2 years, while merely 11.2% had pursued studies for 3 years or longer.

Overall, the international student cohort is predominantly young non-ethnic Chinese undergraduates who demonstrate high academic commitment and limited family cultural ties, though scholarship incentives significantly influence their motivation. The data also highlights their instrumental learning motivation, uneven language proficiency, and relative scarcity of social resources.

## 3 Results

### 3.1 Overall findings of the CLSE scale(CLSES) among international students

Given the diversity of international students in linguistic backgrounds, learning approaches, and cultural adaptation, their cognitive abilities and learning strategies exhibit significant variation depending on their nationalities or prior educational experiences. A systematic analysis of these variations enables a deeper understanding of the distinctive learning profiles and strengths inherent to different student groups.

The four dimensions—cognitive abilities, affective attitudes, learning strategies, and social support—encompass both students’ intrinsic factors (cognitive abilities, affective attitudes, and learning strategies) and external environmental support (social support). This comprehensive framework provides a holistic assessment of the internal and external factors influencing international students’ CLSE. Conducting differential analysis across these dimensions allows researchers to identify which factors exert a more pronounced influence on CLSE. This understanding is imperative for developing targeted interventions to enhance self-efficacy. Furthermore, elucidating inter-individual differences across these dimensions enables educational institutions to formulate precise improvement plans that address the heterogeneous needs of international students from diverse backgrounds, thereby effectively elevating their CLSE.

The reliability and validity test results for the four dimensions of this questionnaire (see Table 3) indicate that all scales meet established research standards. Regarding reliability, the Cronbach’s Alpha coefficients range from 0.710 to 0.744. The social support dimension demonstrates the highest internal consistency (*α* = 0.744), while the affective attitudes dimension exhibits a slightly lower, yet acceptable value (*α* = 0.722). This suggests reliable internal relationships within the item design of each dimension. For validity, all KMO values exceed 0.7. Notably, the learning strategies dimension (0.820) and the social support dimension (0.816) are highly suitable for factor analysis. The affective attitudes dimension (0.781), although the lowest among the dimensions, still meets fundamental requirements. The data demonstrate satisfactory reliability and validity across all dimensions, thereby providing a foundation for subsequent analytical phases.

**Table 3:** Validity and reliability of different dimensions of CLSES for international students.

Dimension	Number of Items	Cronbach’s Alpha	KMO Sample Adequacy Statistic
cognitive abilities	11	0.710	0.800
affective attitudes	17	0.722	0.781
learning strategies	7	0.734	0.820
social support	6	0.744	0.816

### 3.2 Analysis of the differences in CLSE among international students

Cross analysis was performed on 41 scale items corresponding to 18 demographic levels in the questionnaire, and 6 significant differences were found. Subsequently, 4 dimensions in each of the 6 levels were analyzed. The item scores in the following difference analyses were converted to homomorphic scores.

#### 3.2.1 Gender-based analysis

The independent samples *t*-test was performed on the CLSES between male and female international students in this study, with the results presented in Table 4.

**Table 4:** International students’ cognitive abilities disparities based on gender research.

Item	Gender	*n*	M	SD	Levene’s *F*	*t*	Sig.(two-tailed)
56	Male	160	2.74	0.974	0.223	2.232	0.026
	Female	267	2.52	0.998			

The cognitive ability dimension consists of 11 measurement items. Across all 11 items within this dimension, the CLSE scores of female international students were marginally higher than those of male international students. Overall, the scores of international students on the cognitive ability subscale generally ranged from moderate to good levels, where lower scores correspond to higher levels of self-efficacy. A statistically significant gender difference was observed on Item 56 (*p* = 0.026, ≤0.05), indicating that female international students demonstrated significantly greater confidence in their current Chinese language proficiency, and reported that their current Chinese skills adequately meet the requirements of daily life, academic activities, and professional responsibilities.

The affective attitude scale consists of 17 items. With respect to CLSE, male respondents scored no less than 3.0 on items 22 and 27, female respondents scored no less than 3.0 on item 38, male respondents on items 50 and 51, female respondents on item 52, and male respondents on items 58 and 59. These findings suggest that international students’ CLSE on these items falls below the average level. Although male international students exhibited higher self-efficacy than female international students on items 19, 38, 52, and 59, statistically significant differences were only identified on item 27 (*p* = 0.022 ≤ 0.05), item 29 (*p* = 0.02 ≤ 0.05), item 45 (*p* = 0.049 ≤ 0.05), item 50 (*p* = 0.047 ≤ 0.05), and item 54 (*p* = 0.047 ≤ 0.05; see Table 5). To summarize, female international students demonstrate marginally higher levels of confidence in Chinese writing, spoken Chinese, learning certainty, self-assessment of learning efforts, and overall confidence in Chinese language acquisition relative to their male counterparts.

**Table 5:** International students’ affective attitudes disparities based on gender research.

Item	Gender	*n*	Mean	SD	Levene’s *F*	*t*	Sig.(two-tailed)
27	Male	160	3.01	1.052	0.66	2.297	0.022
	Female	267	2.78	0.968			
29	Male	160	2.66	1.132	0.047	2.345	0.020
	Female	267	2.42	0.975			
45	Male	160	2.98	1.058	0.964	1.976	0.049
	Female	267	2.78	0.982			
50	Male	160	3.24	1.119	0.038	1.993	0.047
	Female	267	3.03	1.024			
54	Male	160	2.66	0.990	0.302	1.991	0.047
	Female	267	2.48	0.907			

The learning strategies dimension consists of 7 measurement items. Both male and female international students exhibited above-average CLSE, with female participants consistently demonstrating higher efficacy than male participants. Notably, statistically significant gender differences were only identified on item 46 (*p* = 0.05 ≤ 0.05), indicating that female international students possess markedly higher learning self-efficacy in Chinese learning strategies relative to their male counterparts, as detailed in Table 6.

**Table 6:** International students’ learning strategies disparities based on gender research.

Item	Gender	*n*	M	SD	Levene’s *F*	*t*	Sig.(two-tailed)
46	Male	160	2.78	0.897	0.066	1.969	0.050
	Female	267	2.59	0.951			

Within the 6 items of the social support dimension, no statistically significant gender differences in CLSE were detected across all items, with the only exception of item 44, on which female participants obtained lower scores than male participants. As presented in Table 7, the significant difference between male and female international students observed on item 44 indicates that male international students perceive higher CLSE than their female counterparts when receiving praise for their language proficiency.

**Table 7:** International students’ social support disparities based on gender research.

Item	Gender	*n*	M	SD	Levene’s *F*	*t*	Sig.(two-tailed)
44	Male	160	2.44	1.008	0.722	−0.281	0.779
	Female	267	2.47	1.001			

#### 3.2.2 Age-based analysis

Analyzing the differences in CLSE among international students across various age groups is of paramount importance. Younger students generally exhibit heightened sensitivity to language learning and superior memory capacity, thereby enhancing their adaptability to novel linguistic environments and instructional approaches. Conversely, older students frequently possess more extensive experience in self-regulation and the application of learning strategies. Substantial variations in self-efficacy among distinct age groups are anticipated. To ascertain the presence of significant age-related variations among the surveyed international students, we performed ANOVA analyses on CLSE across three age categories: 18–22 years, 23–27 years, and 27 years and above.

The analysis initially investigates age-related differences in Item 11 of the cognitive abilities dimension. ANOVA results indicated statistically significant between-group differences for Items 34 and 36 (*p* = 0.016 and *p* = 0.040, respectively, with *p* ≤ 0.05), whereas the mean CLSE scores for international students across all three age groups remained marginally above the average level. Subsequent post-hoc LSD tests (see Table 8) revealed that for Item 34, students aged 23–27 years (2.48 ± 0.901) exhibited significantly higher CLSE than both the 18–22 age group (2.74 ± 0.934) and the 27 + age group (2.88 ± 0.833), with the latter group recording the lowest self-efficacy score. For Item 36, the CLSE of international students aged 27 and above (2.52 ± 1.122) was significantly higher than that of the other two age groups (3.03 ± 1.041 and 3.10 ± 1.049).

**Table 8:** Cognitive Ability Disparities among International Students Based on Age Group Research

Item	Source SS	df MS	F	Sig.
34	Between-group 7.017	2 3.508	4.158	
	Within-group 357.761	424 0.844		0.016
	Total 364.778	426		
	(I)Age group	(J)Age group	Mean difference (I-J)	Sig.
	23-27 (2.48±0.901)	18-22 (2.74±0.934) >27 (2.88±0.833)	-0.259 -0.404	0.009 0.045
36 36	Between-group 7.143	2 3.572	3.252	
	Within-group 465.667	424 1.098		0.040
	Total 472.81	426		
	(I)Age group	(J)Age group	Mean difference (I-J)	Sig.
	>27 (2.52±1.122)	18-22 (3.03±1.041) 23-27 (3.10±1.049)	-0.509 -0.583	0.021 0.011

In the dimension of cognitive abilities, international students aged 23 to 27 exhibited significantly higher CLSE in the accurate usage of Chinese vocabulary items 34 and 36 compared to other age groups. Conversely, in addressing pronunciation challenges, those aged 27 and above demonstrated greater CLSE. This divergence suggests that different age cohorts may possess distinct strengths and weaknesses in specific aspects of language acquisition, such as vocabulary application and pronunciation, as well as varied learning experiences.

Subsequently, an ANOVA was performed on the 17 items comprising the affective attitudes dimension across different age groups. The results indicated no statistically significant differences among the items within this dimension. The relevant Chinese language learning self-efficacy data are as follows: for item 22, international students aged 18–22 scored 3.07 (±0.904) and those aged 23–27 scored 3.16 (±0.852); for item 38, scores were 3.03 (±1.080) for the 18–22 age group and 3.16 (±1.046) for the 23–27 age group; for item 50, scores were 3.12 (±1.057) for ages 18–22 and 3.13 (±1.066) for ages 23–27; for item 51, scores were 3.03 (±1.100) for ages 18–22 and 3.10 (±1.026) for ages 23–27; for item 53, individuals over 27 years old scored 3.12 (±0.726); for item 58, the 23–27 age group scored 3.12 (±1.032); and for item 59, scores were 3.03 (±1.215) for ages 18–22 and 3.21 (±1.161) for ages 23–27. The self-efficacy score for this dimension was at a moderately low level, whereas the Chinese language learning self-efficacy across other age groups demonstrated a slightly higher average.

Subsequent ANOVA conducted on the learning strategies dimension (comprising 7 items) revealed that only item 46 exhibited statistically significant differences between groups (*p* = 0.036, ≤0.05). Post-hoc LSD analysis for item 46 indicated a significant difference between international students aged 18–22 (2.75 ± 0.919) and those aged 23–27 (2.50 ± 0.961), with the latter group demonstrating higher CLSE. This finding indicates that students within the 23–27 age group possess stronger self-efficacy regarding Chinese language learning strategies. Across all items within the learning strategies dimension, all age groups exhibited CLSE levels ranging from moderate to high, as detailed in Table 9.

**Table 9:** International students’ learning strategies disparities based on research on age-group.

Item	Source	SS	df	MS	F	Sig.
46	Between-group	5.775	2	2.887	3.345	0.036
	Within-group	365.986	424	0.863		
	Total	371.761	426			
	(I)Age group	(J)Age group		Mean difference(I-J)		Sig.
	18–22 (2.75 ± 0.919)	23–27 (2.5 ± 0.961)		0.246		0.014

Ultimately, an ANOVA was performed on the six items within the social support dimension across the three age groups of international students surveyed. The results revealed no statistically significant intergroup differences. However, the CLSE of international students across all age groups in this dimension was consistently at a moderately high level, as detailed in Table 10. This finding indicates that the CLSE level of international students learning Chinese in China is significantly associated with the following factors: the degree to which Chinese people comprehend their intended meaning, the extent of family support for their Chinese language learning, the influence of learning Chinese on their future planning, their level of acclimatization to living in China, the degree of praise they receive from others regarding their Chinese language ability, and the frequency of school-organized Chinese communication activities.

**Table 10:** International students’ social support disparities based on age-group research.

Item	Age group	M	95% CI for Mean	SD
30	18–22	2.63	(2.52, 2.75)	0.991
	23–27	2.44	(2.26, 2.61)	1.008
	>27	2.32	(2.01, 2.63)	0.748
39	18–22	2.32	(2.20, 2.45)	1.072
	23–27	2.21	(2.02, 2.39)	1.068
	>27	2.00	(1.55, 2.45)	1.080
40	18–22	2.42	(2.29, 2.54)	1.074
	23–27	2.33	(2.14, 2.53)	1.117
	>27	2.16	(1.75, 2.57)	0.987
43	18–22	2.73	(2.62, 2.84)	0.935
	23–27	2.63	(2.45, 2.80)	0.978
	>27	2.80	(2.42, 3.18)	0.913
44	18–22	2.52	(2.40, 2.63)	0.985
	23–27	2.30	(2.11, 2.49)	1.060
	>27	2.64	(2.31, 2.97)	0.810
48	18–22	2.80	(2.69, 2.91)	0.920
	23–27	2.79	(2.62, 2.96)	0.958
	>27	2.80	(2.46, 3.14)	0.816

#### 3.2.3 Analysis based on whether they are ethnic Chinese or not

Ethnic Chinese students, influenced by higher academic expectations and cultural identity from their families and society, tend to demonstrate greater self-efficacy in Chinese language learning. They generally possess a solid linguistic foundation and more educational resources. In contrast, non-ethnic Chinese students often begin learning Chinese from scratch, facing greater language barriers and resource constraints, which may result in lower self-efficacy. Therefore, this study conducted an independent samples *t*-test to compare the CLSE between ethnic Chinese and non-ethnic Chinese international students.

The analysis in the cognitive abilities dimension revealed that only item 35 (non-ethnic Chinese international students: 3.03 ± 1.079) and item 36 (ethnic Chinese: 3.02 ± 1.057; non-ethnic Chinese: 3.02 ± 1.054) showed moderately low CLSE. However, no significant difference was found between ethnic Chinese and non-ethnic Chinese in this dimension.

An independent-samples *t*-test was performed to compare scores on the affective attitudes dimension between ethnic Chinese and non-ethnic Chinese international students. The results revealed statistically significant differences on two items (both *p* ≤ 0.05): item 19 (ethnic Chinese: 2.07 ± 0.896; non-ethnic Chinese: 2.34 ± 0.995, *p* = 0.007) and item 32 (ethnic Chinese: 2.61 ± 1.041; non-ethnic Chinese: 2.87 ± 1.009, *p* = 0.015), as presented in Table 11. This indicates that, by virtue of their ethnic identity, ethnic Chinese international students experience greater confidence in Chinese language learning, resulting in higher CLSE compared to non-ethnic Chinese international students. However, neither group perceives Chinese as an easy language to learn. CLSE scores for both groups across the remaining items all fall within the lower-moderate range, which are detailed as follows: item 50 (ethnic Chinese: 3.07 ± 1.056; non-ethnic Chinese: 3.07 ± 1.056), item 51 (ethnic Chinese: 3.07 ± 1.056; non-ethnic Chinese: 3.07 ± 1.056), item 52 (ethnic Chinese international students: 3.05 ± 1.084; non-ethnic Chinese international students: 3.02 ± 1.089), item 58 (ethnic Chinese international students: 3.01 ± 0.865; non-ethnic Chinese international students: 3.04 ± 1.112), and item 59 (ethnic Chinese international students: 3.07 ± 1.212; non-ethnic Chinese international students: 3.07 ± 1.206).

**Table 11:** International students’ affective attitudes disparities based on research on whether ethnic Chinese or not.

Item	Selection	*n*	M	SD	Levene’s *F*	*t*	Sig.(two tailed)
19	Yes	136	2.07	0.896	0.067	−2.701	0.007
	No	291	2.34	0.995			
32	Yes	136	2.61	1.041	0.088	−2.448	0.015
	No	291	2.87	1.009			

Subsequently, a one-way ANOVA was conducted on the 7 items of the learning strategies dimension across ethnic Chinese and non-ethnic Chinese groups. The results showed that while the CLSE scores for each item were above average, there were no statistically significant differences between the two groups.

Finally, independent sample *t*-tests were conducted on the 6 items of the social support dimension at the levels of ethnic Chinese and Non-ethnic Chinese, showing that the CLSE of each item was higher than the general level. On item 43, which pertained to whether they were accustomed to living in China, the CLSE of ethnic Chinese (2.54±0.981) was significantly higher than that of Non-ethnic Chinese international students (2.78 ± 0.921; *p* = 0.013 ≤ 0.05), as shown in Table 12.

**Table 12:** International students’ social support disparities based on research on whether ethnic Chinese or not.

Item	Selection	*n*	M	SD	Levene’s *F*	*t*	Sig.(two tailed)
43	Yes	136	2.54	0.981	0.038	−2.491	0.013
	No	291	2.78	0.921			

#### 3.2.4 Analysis based on academic level

This study examines international students at three academic levels: university preparatory, undergraduate, and postgraduate. The research demonstrates that analyzing CLSE across educational stages helps clarify developmental characteristics and learning needs. Preparatory students, who face initial language challenges and must achieve specific proficiency benchmarks within a defined timeframe, may exhibit self-efficacy influenced by foundational language skills. Undergraduates, with established language proficiency, tend to prioritize academic and career development goals. Postgraduates, typically possessing advanced language competence and higher academic demands, show self-efficacy more closely tied to research and professional advancement. To validate these hypotheses, the study conducted ANOVA analyses across all dimensions for each educational level.

An ANOVA analysis of the academic levels of international students was conducted from the cognitive abilities dimension, revealing significant between-group differences in items 25, 36, and 57. Subsequently, post-hoc LSD tests were performed (*p* = 0.018, 0.028, 0.032 ≤ 0.05).

As shown in Table 13, in Item 25, preparatory students (2.20 ± 0.848) demonstrated significantly higher CLSE compared to undergraduates (2.61 ± 1.006) and postgraduates (2.56 ± 0.989). This indicates that preparatory students exhibit greater confidence in the listening section than other academic levels of international students (*p* = 0.005, 0.032 ≤ 0.05). In Item 36, preparatory students (2.75 ± 0.886) showed significantly higher CLSE than postgraduates (3.22 ± 1.063). Notably, the postgraduates’ self-efficacy in Item 36 consistently exceeded 3.0, indicating a moderately low level of confidence in Chinese pronunciation (*p* = 0.008 ≤ 0.05). For Item 57, preparatory students (2.69 ± 0.690) demonstrated significantly higher CLSE than undergraduates (2.96 ± 0.834). This suggests that preparatory students studying in China are more confident in their overall Chinese proficiency compared to undergraduates, with greater belief in their language skills ranking higher within their class, reflecting higher CLSE (*p* = 0.026 ≤ 0.05).

**Table 13:** Cognitive Ability Disparities among International Students Based on Education Level Research

Item	Source	SS	df	MS	F	Sig.
25	Between-group	7.886	2	3.943	4.079	0.018
	Within-group 409.88		424 0.967			
	Total 417.766		426			
	(I) Education level		(J)Education level		Mean difference (I-J)	Sig.
	preparatory		undergraduate (2.61±1.006)		-0.415	0.005
	(2.20±0.848)		postgraduate (2.56±0.989)		-0.357	0.032
36	Between-group 7.935		2 3.967		3.619	
57	Within-group Total		464.875 472.81		424 1.096 426	0.028
	(I)Education level preparatory (2.75±0.886)		(J)Education level postgraduate (3.22±1.063)		Mean difference (I-J) -0.471	Sig. 0.008
	Between-group		4.618		2 2.309	3.462
57 57	Within-group Total		282.759 287.377		424 0.667 426	0.032
	(I) Education level preparatory (2.69±0.69)		(J)Education level undergraduate (2.96±0.834)		Mean difference (I-J) -0.269	Sig. 0.026

The ANOVA analysis of affective attitudes among international students of different academic levels showed that there were significant differences among the groups of affective attitudes (*p* = 0.011, 0.023, 0.013, 0.012, 0.001, 0.000 ≤ 0.05).

As shown in Table 14, preparatory students demonstrate significantly higher CLSE acquisition compared to international students at other academic levels. Item 22 reveals a notable difference: preparatory students (2.8 ± 0.621) exhibit significantly higher self-efficacy (*p* = 0.029, 0.003 ≤ 0.05) than undergraduates (3.08 ± 0.914) and postgraduates (3.25 ± 0.878), while undergraduates andpostgraduates show relatively low self-efficacy in this item. This indicates that preparatory international students possess greater self-efficacy in Chinese task performance than their peers at other academic levels. Item 24 further demonstrates that preparatory students (2.40 ± 0.807) exhibit significantly higher self-efficacy (*p* = 0.021 ≤ 0.05) than undergraduates (2.72 ± 0.968) in Chinese language proficiency, indicating a marked advantage in test-taking confidence among preparatory students. In item 45, preparatory students (2.51 ± 0.767) demonstrated significantly higher CLSE than undergraduates (2.86 ± 1.041) and postgraduates (3.01 ± 1.026), with postgraduates showing a marginally lower self-efficacy than the average level (*p* = 0.018, 0.003 ≤ 0.05). This indicates that international students’ confidence in Chinese learning decreases with higher academic qualifications. In item 53, preparatory students (2.60 ± 0.760) maintained the highest CLSE, which was significantly higher than undergraduates (2.99 ± 0.884; *p* = 0.003 ≤ 0.05), suggesting that undergraduates exhibit significantly greater test anxiety in Chinese. In item 58, preparatory students (2.49 ± 0.814) showed significantly higher self-efficacy than other academic levels (undergraduates: 3.06 ± 1.125; postgraduates: 3.19 ± 1.176) (*p* = 0.001, 0.000 ≤ 0.05). Furthermore, both undergraduates and postgraduates demonstrated relatively low to moderate levels of reading self-efficacy in Chinese. Item 59 revealed that preparatory students (2.51 ± 0.960) demonstrated significantly higher CLSE than undergraduates (3.08 ± 1.238) and postgraduates (3.36 ± 1.138) (*p* = 0.001, 0 ≤ 0.05). Notably, undergraduates (3.08 ± 1.238) also showed significantly higher self-efficacy than postgraduates (3.36 ± 1.138) (*p* = 0.045, 0 ≤ 0.05). These findings indicate significant differences in Chinese writing confidence among international students across the three academic levels, with undergraduates and postgraduates demonstrating lower self-efficacy than the general population.

**Table 14:** International Students’ Affective Attitudes Disparities Based on Education Level Research

Item	Source SS		df MS	F	Sig.
22	Between-group 7.027 7.027		2 3.513	4.599	
	Within-group 323.938		424 0.764		0.011
	Total 330.965		426		
	(I) Education level		(J)Education level	Mean difference (I-J)	Sig.
	preparatory (2.80±0.621)		undergraduate (3.08±0.914) postgraduate (3.25±0.878)	-0.284 -0.447	0.029 0.003
24	Between-group Within-group Total	6.83 378.444 385.274	2 3.415 424 0.893 426	3.826	0.023
	(I) Education level preparatory (2.40±0.807)		(J)Education level undergraduate (2.72±0.968)	Mean difference (I-J) -0.324	Sig. 0.021
45	Between-group Within-group Total	8.923 429.484 438.407	2 4.462 424 1.013 426	4.405	0.013
(I) Education level preparatory (2.51±0.767)			(J)Education level undergraduate (2.86±1.041) postgraduate (3.01±1.026)	Mean difference (I-J) -0.353 -0.501	Sig. 0.018 0.003
Between-group Within-group Total	6.9 326.894 333.794		2 3.45 424 0.771 426	4.475	0.012
(I) Education level preparatory (2.60±0.760)			(J)Education level undergraduate (2.99±0.884) postgraduate (3. 19±1. 176)	Mean difference (I-J) -0.385	Sig. 0.003
Between-group Within-group Total	18.441 515.474 533.916		2 9.221 424 1..216 426	7.584	0.001
(I) Educaiton level preparatory			(J)Education level undergraduate	Mean difference (I-J) -0.567	Sig. 0.001
	(2.49±0.814)		(3.06±1.125) postgraduate (3.19±1.176)	-0.695	0.000
59	Between-group 25.536		2 12.768	9.108	
	Within-group 594.357		424 1.402		0.000
	Total 619.892		426		
	(I) Education level		(J)Education level	Mean difference (I-J)	Sig.
	Preparatory (2.51±0.960)		undergraduate (3.08±1.238)	-0.571 -0.852	0.001 0.000

undergraduate (3.08±1.238)	postgraduate (3.36±1.138)	-0.281	0.045

The ANOVA analysis of the learning strategies dimension revealed significant between-group differences (*p* = 0.029 and 0.033, ≤0.05) for items 46 and 47. Post-hoc LSD analysis of items 46 and 47 showed that postgraduates (2.48 ± 0.948) demonstrated significantly higher CLSE than undergraduates (2.75 ± 0.943) in learning strategies, indicating that postgraduates have developed established study methods through years of academic experience. However, preparatory students (2.25 ± 0.726) exhibited significantly higher CLSE than undergraduates (2.57 ± 0.988) in seeking advice from others or consulting materials (*p* = 0.023, ≤0.05), suggesting that postgraduates tend to rely more on self-study while preparatory students prefer seeking external assistance (see Table 15).

**Table 15:** Disparities in learning strategies among international students, based on research on educational levels.

Item	Source	SS	df	MS	*F*	Sig.
46	Between-group	6.138	2	3.069	3.559	0.029
	Within-group	365.623	424	0.862		
	Total	371.761	426			
	(I) Education level	(J) Education level		Mean difference (I-J)		Sig.
	Preparatory (2.48 ± 0.948)	Undergraduate (2.75 ± 0.943)		−0.265		0.016
47	Between-group	6.158	2	3.079	3.43	0.033
	Within-group	380.545	424	0.898		
	Total	386.703	426			
	(I) Education level	(J) Education level		Mean difference (I-J)		Sig.
	Preparatory (2.25 ± 0.726)	Undergraduate (2.57 ± 0.988)		−0.320		0.023

Table 16 presents the difference analysis on the dimension of social support. The ANOVA analysis revealed significant differences between groups for items 30, 39, and 40 (*p* = 0.004, 0.048, 0.042 ≤ 0.05). Post-hoc LSD analysis showed that when Chinese people could quickly understand what they wanted to express, the CLSE of preparatory (2.24 ± 0.719) and postgraduates (2.42 ± 0.945) was significantly higher than that of undergraduates (2.67 ± 1.030) (*p* = 0.003, 0.033 ≤ 0.05). The families of postgraduates (2.04 ± 1.050) were more supportive of their Chinese learning than those of undergraduates (2.35 ± 1.099) (*p* = 0.014 ≤ 0.05). However, it is noteworthy that preparatory (2.09 ± 0.888) perceived that learning Chinese would significantly affect their future plans more than undergraduates (2.47 ± 1.108; *p* = 0.019 ≤ 0.05).

**Table 16:** Disparities in social support among international students, based on research on educational levels.

Item	Source	SS	df	MS	*F*	Sig.
30	Between-group	10.86	2	5.43	5.692	0.004
	Within-group	404.485	424	0.954		
	Total	415.344	426			
	(I) Education level	(J) Education level		Mean difference (I-J)		Sig.
	Preparatory (2.24 ± 0.719)	Undergraduate (2.67 ± 1.030)		−0.433		0.003
		Postgraduate (2.42 ± 0.945)				
				−0.246		0.033
39	Between-group	6.971	2	3.486	3.059	0.048
	Within-group	483.057	424	1.139		
	Total	490.028	426			
	(I) Education level	(J) Education level		Mean difference (I-J)		Sig.
	Postgraduate (2.04 ± 1.050)	Undergraduate (2.35 ± 1.099)		−0.311		0.014
40	Between-group	7.41	2	3.705	3.2	0.042
	Within-group	490.885	424	1.158		
	Total	498.295	426			
	(I) Education level	(J) Education level		Mean difference (I-J)		Sig.
	Preparatory (2.09 ± 0.888)	Undergraduate (2.47 ± 1.108)		−0.375		0.019

#### 3.2.5 Analysis based on whether students have learned foreign languages except for Chinese

Generally, students with prior foreign language experience have typically undergone a language acquisition process from unfamiliarity to proficiency, mastering fundamental learning methods and techniques. This background enables them to better handle new language challenges, which may lead to higher CLSE. Moreover, their experience in mastering other foreign languages can enhance the confidence of international students, making them more confident and patient when learning Chinese. Therefore, we conducted an independent samples *t*-test on the CLSES based on the criterion of whether international students have studied foreign languages other than Chinese.

First, we conducted a one-way ANOVA on the dimension of cognitive abilities to examine whether international students had studied foreign languages other than Chinese. The results showed that international students who had studied foreign languages other than Chinese (2.77 ± 1.053) had significantly higher CLSE (*p* = 0.05) compared to those who had not (3.19 ± 1.083). This indicates that students with prior foreign language learning experience demonstrated greater confidence in mastering Chinese grammar. Similar findings were observed in item 36: students who had studied foreign languages other than Chinese (2.87 ± 1.077) showed significantly higher CLSE (*p* = 0.05) than those who had not (3.14 ± 1.019). Furthermore, these students exhibited significantly greater confidence in Chinese pronunciation learning (*p* = 0.007 ≤ 0.05) compared to their non-experienced counterparts. See Table 17.

**Table 17:** International students’ cognitive abilities disparities based on whether they learn foreign languages other than Chinese or not.

Item	Whether learn any other foreign languages before learning Chinese or not	n	M	SD	Levene’s *F*	t	Sig. (two-tailed)
35	No	235	3.19	1.083	0.463	4.042	0.000
	Yes	192	2.77	1.053			
36	No	235	3.14	1.019	0.562	2.702	0.007
	Yes	192	2.87	1.077			

From the perspective of cognitive abilities, Table 17 data indicates that international students who have studied other foreign languages score lower in Chinese grammar and pronunciation than those who have not, suggesting they have higher self-efficacy in these areas. This may be because they have acquired relevant language knowledge and learning strategies during their language studies, enabling them to handle the challenges of Chinese grammar and pronunciation with greater confidence.

Subsequently, a one-way ANOVA was conducted on the 17 items of the affective attitudes dimension regarding whether international students had studied foreign languages other than Chinese. The results revealed significant differences in items 38, 50, 58, and 59. For these items, international students who had studied foreign languages other than Chinese (2.94 ± 1.034, 2.91 ± 1.003, 2.75 ± 1.107, 2.92 ± 1.127) demonstrated significantly higher self-efficacy in Chinese learning compared to those who had not (3.17 ± 1.099, 3.27 ± 1.086, 3.23 ± 1.085, 3.2 ± 1.256) (*p* = 0.033,0, 0, 0.017 ≤ 0.05), as shown in Table 18.

**Table 18:** International students’ affective attitudes disparities based on research on whether learning foreign languages other than Chinese or not.

Item	Whether learn any other foreign languages before learning Chinese or not	n	M	SD	Levene’s *F*	t	Sig. (two-tailed)
38	No	235	3.17	1.099	0.236	2.144	0.033
	Yes	192	2.94	1.034			
50	No	235	3.27	1.086	0.019	3.543	0.000
	Yes	192	2.91	1.003			
58	No	235	3.23	1.085	0.899	4.504	0.000
	Yes	192	2.75	1.107			
59	No	235	3.20	1.256	0.001	2.391	0.017
	Yes	192	2.92	1.127			

In the affective attitudes dimension, international students who had studied languages other than Chinese demonstrated higher CLSE than those who had not. This was particularly evident in items measuring classroom tension, concerns about learning outcomes, and confidence in Chinese reading and writing. The findings suggest that these students possess stronger cognitive self-efficacy in Chinese learning, making them less prone to negative emotions stemming from cognitive challenges in the learning process.

The analysis conducted an independent samples *t*-test on the 7 items of learning strategies regarding international students’ exposure to foreign languages other than Chinese. The results showed that international students who had studied foreign languages other than Chinese (2.36 ± 0.893) demonstrated significantly higher CLSE (2.60 ± 0.988) compared to those who had not (*p* = 0.011, ≤0.05), as detailed in Table 19. This indicates that students with prior foreign language exposure are more likely to seek help or consult resources when encountering difficulties, reflecting a stronger drive for knowledge acquisition.

**Table 19:** International students’ learning strategies disparities based on research on whether learning foreign languages other than Chinese.

Item	Whether learn any other foreign languages before learning Chinese ot not	n	M	SD	Levene’s *F*	*t*	Sig. (two-tailed)
47	No	235	2.60	0.988	0.13	2.567	0.011
	Yes	192	2.36	0.893			

Finally, the social support dimension did not show significant differences in terms of whether international students had studied foreign languages other than Chinese. This indicates that the study of foreign languages other than Chinese was not correlated with the social support received by international students.

#### 3.2.6 Analysis based on the duration of Chinese learning

A one-way ANOVA was conducted to examine differences in CLSE on the cognitive abilities dimension among international students grouped by their duration of Chinese language study. The analysis revealed a statistically significant inter-group difference only for item 36 (*p* = 0.002). Subsequent LSD post-hoc tests for this item (detailed in Table 20 below) indicated that, within the cognitive abilities dimension, the CLSE score for students who had studied Chinese for two to 3 years (3.50 ± 1.072) was significantly lower than the scores for those in the following duration groups: less than 6 months (2.72 ± 1.015; *p* < 0.001), 6 months to 1 year (2.86 ± 1.003; *p* = 0.001), 1–2 years (2.98 ± 1.000; *p* = 0.003), and two to 3 years (3.10 ± 1.091; *p* = 0.022). Furthermore, within the latter comparative pairing, the CLSE of students in the two-to-three-year group (3.10 ± 1.091) was itself significantly lower than that of students in the less-than-six-months group (2.72 ± 1.015; *p* = 0.037). These findings suggest that students with less than 6 months of Chinese study exhibit greater confidence in their pronunciation abilities compared to their counterparts who have studied for 2–3 years.

**Table 20:** International students’ cognitive abilities disparities based on research on Chinese learning duration.

Item	Source	SS	df	MS	F	Sig.
36	Between-group	18.171	2	4.543	4.216	0.002
	Within-group	454.64	424	1.077		
	Total	472.81	426			
	(I) Chinese learning duration	(J)Chinese learning duration		Mean difference(I-J)		Sig.
	Within 6 months (2.48 ± 0.948)	>3 year (3.50 ± 1.072)		−0.777		0.000

	0.5–1 year (2.86 ± 1.003)			−0.641		0.001
	2 years (2.98 ± 1.000)			−0.523		0.003
	2–3 years (3.10 ± 1.091)			−0.405		0.022
	Within 6 months (2.72 ± 1.015)	2–3 years (3.10 ± 1.091)		−0.372		0.037

An ANOVA analysis of affective attitudes on international students’ Chinese learning duration revealed significant differences between items 29, 45, 53, 58, and 59 (*p* = 0.048, 0.038, 0.038, ≤0.05). Subsequent LSD post-hoc tests (Table 21) showed that in item 29, students with 6 months of Chinese learning experience (2.09 ± 0.929) demonstrated significantly higher self-efficacy than those with 6 months to 1 year (2.51 ± 1.003), 1 to 2 years (2.53 ± 1.143), 2–3 years (2.60 ± 0.965), and over 3 years (2.65 ± 1.062). This indicates that beginners are more willing to demonstrate their spoken Chinese proficiency compared to long-term learners (*p* = 0.0260, 0.012, 0.004, 0.009, ≤0.05).

**Table 21:** International students’ learning strategies disparities based on research on Chinese learning duration.

Item	Source	SS	df	MS	F	Sig.
30	Between-group	11.266	2	2.817	2.941	0.020
	Within-group	404.078	424	0.958		
	Total	415.344	426			
	(I) Chinese learning duration	(J) Chinese learning duration		Mean difference (I-J)		Sig.
	Within 6 months	0.5–1 year (2.54 ± 0.963)		−0.368		0.042
		1–2 years (2.55 ± 0.921)		0.377		0.025
		2–3 years (2.74 ± 1.005)		−0.568		0.001

In item 45, international students with 2–3 years (2.97 ± 1.058) and over 3 years (3.10 ± 0.973) of Chinese learning experience showed significantly lower self-efficacy in Chinese learning compared to those with 6–12 months (2.63 ± 0.995) of experience (*p* = 0.02, 0.01 ≤ 0.05).

For Item 53, the Chinese learning self-efficacy score of international students with a Chinese learning duration of no more than 6 months (2.70 ± 0.805) was significantly higher than that of students with a learning duration of 1–2 years (3.02 ± 0.900), with the difference reaching statistical significance (*p* = 0.033, *p* ≤ 0.05). Additionally, the self-efficacy scores of international students with a Chinese learning duration ranging from 6 months to 1 year (2.71 ± 0.775) were significantly higher than those of students with a learning duration of 1–2 years (3.02 ± 0.900) and 2–3 years (2.99 ± 0.890) (*p* = 0.012, *p* = 0.024, all *p* ≤ 0.05). These results indicate that the CLSE of international students with a Chinese learning duration of less than 1 year is significantly higher than that of students with a learning duration of 1–3 years.

In item 58, international students who had studied Chinese for 6 months or less (2.68 ± 1.065) demonstrated significantly higher self-efficacy in Chinese learning compared to those who studied for 2–3 years (3.08 ± 1.100) and those who studied for 3 years or more (3.69 ± 1.035; *p* = 0.032, ≤0.050). Similarly, students who studied Chinese for 6 months to 1 year (2.65 ± 1.055) showed significantly higher self-efficacy than those who studied for one to 2 years (3.04 ± 1.111), 2–3 years (3.08 ± 1.100), and 3 years or more (3.69 ± 1.035; *p* = 0.014, 0.007, ≤0.05). Furthermore, students who studied Chinese for one to 2 years (3.04 ± 1.111) exhibited significantly higher self-efficacy than those who studied for 3 years or more (3.69 ± 1.035) (*p* = 0 ≤ 0.05). International students with 2–3 years of Chinese language study (3.08 ± 1.100) demonstrated significantly higher self-efficacy in Chinese reading compared to those with over 3 years of study (3.69 ± 1.035) (*p* = 0.001, ≤0.05). Except for those with study durations between 6 months and 1 year, most international students exhibited moderately low confidence in Chinese reading. This phenomenon may stem from the increasing complexity of Chinese characters as their proficiency deepens, thereby elevating reading difficulty and consequently reducing self-efficacy over time.

For Item 59, the Chinese learning self-efficacy scores of international students with a learning duration of less than 6 months (2.64 ± 1.092) were significantly higher than those of students with a learning duration of 1–2 years (3.07 ± 1.198) and 2–3 years (3.16 ± 1.223; *p* = 0.033, 0.01 ≤ 0.05). In contrast, the self-efficacy scores of international students with a learning duration exceeding 3 years (3.65 ± 1.021) were significantly lower than those of students with shorter learning durations (*p* = 0.004, 0.016 ≤ 0.05). The above empirical results show that with the improvement of Chinese proficiency, the perceived difficulty of Chinese character writing increases for international students, due to the complex strokes and multiple similar character components of Chinese characters. This further leads to a gradual decrease in the learners’ Chinese learning self-efficacy as learning duration extends.

In the dimension of learning strategies, there was no significant difference in the duration of Chinese learning among international students across groups on the 7 items. However, in the dimension of social support, item 30 showed a significant difference between groups (*p* = 0.020 ≤ 0.05). For item 30, an LSD post-hoc test was conducted, and the CLSE of international students who had studied Chinese for less than 6 months (2.17 ± 0.940) was significantly higher than that of those who had studied Chinese for 6 months to 1 year (2.54 ± 0.963), 1 year to 2 years (2.55 ± 0.921), and 2 years to 3 years (2.74 ± 1.005) (*p* = 0.042, 0.025, 0.001 ≤ 0.05), as shown in Table 21 below. This indicates that as the duration of Chinese learning increases, international students’ CLSE in responding quickly to Chinese speakers in China decreases. This may be because even if Chinese speakers can quickly understand their meaning, international students feel that their expression is not accurate enough.

### 3.3 Summary

Through the analysis of the individual differences of the learners, we can understand that there are significant differences in the gender, age, whether ethnic Chinese, educational level, whether they have studied other foreign languages besides Chinese and the length of time of learning Chinese among the Chinese learners in China. The main individual differences are reflected in the following aspects:

Firstly, female international students demonstrate slightly higher self-efficacy in language acquisition than their male counterparts. This finding aligns with Bandura’s self-efficacy theory, which posits that individuals build self-efficacy through observing others’ success, social reinforcement, and emotional states. Notably, female in language learning are more likely to benefit from positive feedback and encouragement.

Secondly, Chinese learners aged 23–27 and older demonstrated higher cognitive abilities than younger groups, indicating that older learners rely more on their prior knowledge. In contrast, 18–22-year-olds, while showing greater confidence in their learning strategies, exhibited slightly higher anxiety levels in the emotional dimension compared to other age groups.

Thirdly, ethnic Chinese international students demonstrated higher self-efficacy in Chinese language learning regarding affective attitudes and social support than their non-ethnic counterparts. No significant differences were observed between ethnic and non-ethnic Chinese students in cognitive abilities and learning strategies. These findings align with the established patterns of how cultural background and social support influence CLSE.

Fourthly, the self-efficacy of preparatory students in Chinese learning in four dimensions of cognitive abilities, affective attitudes, learning strategies and social support is the highest among the three academic levels, which may be closely related to the novice effect.

Fifth, international students with prior foreign language experience demonstrate higher CLSE, indicating that their previous language learning experiences, strategies, and sense of achievement significantly contribute to their new language acquisition.

Sixth, regarding the duration of Chinese language learning, learners ‘self-efficacy generally follows an upward trend initially followed by a decline. Beginners exhibit higher self-efficacy in early stages, but as learning difficulty increases and their understanding of the language’s complexity becomes clearer, this self-efficacy may slightly decrease before stabilizing. This pattern could be attributed to language learning fatigue.

## 4 Discussion

### 4.1 Existing issues

This study analyzed the current status of international students ‘CLSE through data analysis, examining the impact of several different aspects and background factors on their self-efficacy, including gender, age group, whether they are ethnic Chinese or not, educational level, duration of Chinese learning, whether they have learned foreign languages other than Chinese.

According to the statistical results of the total table, the mean value of international students’ self-efficacy in learning Chinese in this survey is at a medium-high level (110.8/41 ≈ 2.7). In general, international students still believe that they can learn Chinese well in university in China.

#### 4.1.1 Problems in the dimensions of the scale

The learners showed different levels of CLSE in four dimensions: cognitive abilities, affective attitudes, learning strategies and social support.

The average score of international students in China on the cognitive ability dimension of CLSE is at a moderate level (2.73), suggesting that the majority of respondents hold a positive confidence evaluation of their overall Chinese language proficiency, including practical application competence and Chinese language thinking ability. However, a subset of respondents still exhibits low confidence in their mastery of Chinese grammatical structures and pronunciation.

Among the four dimensions examined in this study, the CLSE score of the affective attitudes dimension is the lowest (*M* = 2.82). A large proportion of respondents experience difficulties in Chinese character writing and reading, which are frequently accompanied by learning anxiety, and consequently undermine learners’ Chinese language learning confidence. Such negative affective attitudes can further reduce learners’ learning motivation and persistence during Chinese language acquisition.

Regarding the learning strategies dimension, the CLSE of learners reaches a moderate-to-high level (*M* = 2.54). This result indicates that a large proportion of international students perceive themselves as having relatively strong capabilities in developing Chinese learning schedules, managing study time, and adopting effective learning methods, which reflects their comparatively high learning efficiency.

Across all Chinese language learning dimensions, international students obtained the highest self-efficacy score on the social support dimension (*M* = 2.53). Most respondents self-reported that they received substantial support from their Chinese language teachers, peers, and family members during the learning process. This support helps foster learners’ sense of learning security and exerts a positive effect on their Chinese learning outcomes.

#### 4.1.2 Problems with the mean values of items

Further descriptive analysis of the item-level mean scores of the overall scale yielded consistent findings. On the five-point rating scale adopted in this study, eight items exhibited mean scores equal to or higher than 3.0, indicating that these items collectively contribute to the decline in the average level of CLSE among the international student sample. As presented in Table 22, of the eight aforementioned items, six are classified under the affective attitude dimension, and the remaining two are categorized within the cognitive ability dimension.

**Table 22:** Items with a mean below the median.

Item	M	SD	Dimension
50	3.11	1.064	
22	3.08	0.881	
38	3.07	1.075	Affective attitudes
59	3.07	1.206	
51	3.03	1.086	
36	3.02	1.054	Cognitive abilities
58	3.01	1.120	Affective attitudes
35	3.00	1.089	Cognitive abilities

##### 4.1.2.1 Affective attitudes dimension

All items under the affective attitudes dimension yielded mean scores higher than 3.0, which are specifically: anxiety about learning outcomes (item 50), self-doubt concerning academic performance (item 22), anxiety and apprehension towards attending Chinese language courses (item 38), aversion to Chinese character writing (item 59), low self-evaluation of learning achievements (item 51), and difficulty in reading Chinese characters (item 58). These results reflect the subjective perceptions of international students, rather than empirical evidence for the existence of actual deficiencies in their Chinese language proficiency. The core issue is most likely that the negative emotional experiences encountered by these students during Chinese language learning are significantly more prominent than the actual gaps in Chinese language ability perceived by learners.

##### 4.1.2.2 Dimension of cognitive abilities

Only two items under the cognitive abilities dimension obtained mean scores higher than 3.0, which are specifically “Perceiving Chinese pronunciation as difficult” (item 36) and “Perceiving Chinese grammar as difficult” (item 35). These difficulties stem from international students’ lack of familiarity with Chinese grammatical rules and pronunciation patterns, which differ substantially from the corresponding linguistic rules of their native languages. The core cause is most likely the insufficient mastery of Chinese linguistic elements including grammar, pronunciation and vocabulary among the respondents.

#### 4.1.3 Problems in the duration of Chinese learning

In the analysis of Chinese language learning duration, an unexpected finding emerged: Among all groups divided by Chinese learning duration, international students with more than 3 years of Chinese language learning obtained the lowest average item score (*M* = 2.97) across the four research dimensions of affective attitudes. This result indicates that prolonged learning experience will increase the perceived difficulty of the target language, and reduce learners’ learning self-confidence. In comparison, international students with 1 to 2 years of Chinese learning experience obtained the highest average item score (*M* = 2.17) in the cognitive ability dimension, which exceeds the average scores of all other groups.

### 4.2 Causes of the issue

#### 4.2.1 Individual reasons

##### 4.2.1.1 Female international students demonstrate superior capabilities in emotional management and the application of learning strategies

The results of the gender analysis show that in the females’ CLSE, is slightly higher than that of the male.

Consistent with the findings of Liu et al. (2012), female learners are more inclined to adopt cognitive strategies such as cognitive reappraisal to regulate emotions, while male learners mainly rely on automatic emotion regulation approaches. This indicates that when encountering negative emotional experiences, male learners tend to adopt more direct response modes, rather than processing and regulating emotions through deliberate cognitive analysis. Empirical evidence shows that female learners achieve higher rationality in emotion regulation by analyzing their current learning situation and mobilizing cognitive abilities, which is significantly different from the more immediate response mode adopted by male learners. Li and Wang (2010) obtained consistent research conclusions, pointing out that male learners typically present field-independent cognitive styles and have comparative advantages in writing and grammar-related tasks in second language learning, while female learners usually present field-dependent cognitive styles and show higher learning proficiency in language acquisition and social interaction activities. In addition, female learners generally tend to have more extroverted learning styles, while male learners tend to have more introverted learning styles. The above research conclusions also provide a theoretical explanation for the significantly higher CLSE of female learners compared with male learners in both the cognitive ability dimension and the affective attitude dimension.

The results regarding the gender difference dimension of learning strategies obtained in this study are consistent with the conclusions of existing research by Wang (2015) and Xia (2022), which confirms that female learners show higher initiative when using various learning strategies, and the frequency of strategy application is also significantly higher.

The higher CLSE of female international students relative to male international students in the social support dimension can be mainly attributed to gender-specific socialization processes and gender role expectations. In most cultural contexts, women are often socialized to rely on relational networks (including family systems and social circles) to obtain resources and support. This socialized dependence tendency enables women to access learning resources such as language exchange partners, study groups and mentorship guidance more conveniently. Traditional gender role expectations also further reinforce this phenomenon. For example, when a society generally constructs the gender image of women as “gentle” and “cooperative,” women will have stronger intrinsic motivation to seek and accept external help, so as to improve their CLSE level. In contrast, traditional gender norms require men to maintain higher independence, which may lead men to prefer to independently resolve academic difficulties rather than actively seek external help. The higher level of social support obtained by women explains why they show higher CLSE in the social support dimension.

##### 4.2.1.2 Younger international students generally have weaker learning motivation

The influence of age differences on CLSE is a complex research topic that involves multidisciplinary inquiry across psychology and education. Within the context of Chinese language learning research, when categorizing learners by age groups, existing evidence indicates that, relative to younger learners, older learners generally exhibit higher levels of self-efficacy in the process of language acquisition.

The influence of age differences on CLSE can be interpreted from multiple theoretical perspectives. Erikson’s psychosocial development theory indicates that identity uncertainty among younger learners may lead to insufficient learning confidence, while older learners with relatively stable self-identity exhibit higher levels of self-efficacy. Gardner.et al.’s (1985) language learning motivation theory holds that older learners generally have stronger instrumental motivation, which promotes higher levels of engagement and persistence in Chinese language acquisition. Oxford’s (1990) research on language learning strategies further demonstrates that older learners with accumulated learning experience can use language learning strategies more effectively, which in turn improves their CLSE. The above theoretical perspectives and empirical findings jointly support the conclusion that older learners generally show higher CLSE than younger learners. This intergroup difference does not originate solely from age itself, but is the joint outcome of multiple factors including psychological development status, learning motivation level and accumulated learning experience. This conclusion has important guiding implications for Chinese language teaching practice. By deeply understanding the learning needs and individual characteristics of learners of different age groups, teachers can develop more targeted and effective instructional strategies, and ultimately improve the self-efficacy and learning outcomes of learners across all age groups.

##### 4.2.1.3 The advantages of Chinese language learning for ethnic Chinese international students supported by family culture

The advantage of ethnic Chinese international students in Chinese language learning originates from their early exposure to the Chinese language in daily contexts. Although some ethnic Chinese students mainly use other languages in their native linguistic environment, the cultural inheritance embedded in their family background endows them with a certain foundational cognition of the Chinese language and Chinese culture. By contrast, non-ethnic Chinese students lack the support of a family language background, but they can bridge the cognitive gap through systematic school instruction and effective mastery of learning strategies. Consequently, no significant difference is observed between ethnic Chinese and non-ethnic Chinese students in terms of language cognitive ability and learning strategy application.

Nevertheless, in the dimension of affective attitude, ethnic Chinese students generally show more positive affective tendencies toward the Chinese language. This discrepancy not only originates from differences in cultural identity, but also stems from intrinsic motivation: ethnic Chinese students generally regard Chinese language learning as an opportunity to deepen the connection with their ancestral culture, and such intrinsic motivation enables them to achieve higher levels of concentration and deeper engagement in Chinese learning, thereby enhancing their self-efficacy in the process of language acquisition. In contrast, non-ethnic Chinese students, especially those from cultural backgrounds that have little connection with Chinese culture, often lack such inherent affective connection. Accordingly, it is more difficult for them to form cultural resonance in the learning process, and the frequent frustration they experience will in turn damage their learning self-efficacy.

In terms of social support, ethnic Chinese students generally have access to richer social resources within the Chinese learning context compared to non-ethnic Chinese students. They often receive support from family members, relatives and the local ethnic Chinese community, which enables them to adapt to Chinese cultural and daily life more smoothly. This level of social support not only facilitates faster adaptation to the Chinese language learning environment, but also provides learners with psychological security and a sense of belonging, thereby effectively enhancing their Chinese language learning confidence and learning motivation. By contrast, non-ethnic Chinese students often lack equivalent supporting systems when studying in a foreign country, and are more likely to experience culture shock and feelings of isolation, which further negatively affects their emotional stability during the Chinese language learning process.

Ethnic Chinese students demonstrate significantly higher CLSE than their non-ethnic Chinese counterparts, primarily due to their stronger emotional engagement and social support. While no significant differences were observed in cognitive abilities and learning strategies between the two groups, the cultural background and social support systems of ethnic Chinese students provide them with greater advantages in emotional investment and social adaptability, which substantially enhances their self-efficacy.

##### 4.2.1.4 Novice effect and strong target focusing of Chinese beginners

This phenomenon can be explained from multiple dimensions, namely the novice effect, concentration on learning objectives, supportive learning environment, and targeted curriculum design.

The novice effect refers to the phenomenon that beginners will gain higher self-confidence and learning motivation in the early stage of learning, driven by rapid learning progress and initial learning outcomes, which in turn promotes the improvement of their self-efficacy. This phenomenon is particularly significant among preparatory students. For learners who start learning Chinese from scratch or with a limited Chinese foundation, their Chinese proficiency usually achieves substantial improvement in the initial stage: they can quickly show clear progress by mastering basic vocabulary, simple grammar, or being able to conduct daily basic conversations. Such rapid progress brings learners a strong sense of achievement, which further enhances their level of self-efficacy. By contrast, international students enrolled in undergraduate or postgraduate programs have generally completed this stage of rapid improvement, and their overall learning progress has entered a stable stage. Accordingly, their self-efficacy cannot maintain the rapid growth rate observed in preparatory class students.

The single-focused characteristic of learning objectives allows preparatory course students to devote all their attention to Chinese language acquisition. Unlike undergraduate or postgraduate students who are required to complete professional course learning concurrently, the core task of preparatory students is to lay a solid foundation for Chinese language competence. This unitary focus enables them to concentrate their efforts on Chinese learning, and continuously enhances their level of self-efficacy with the gradual improvement of their language proficiency. Yu et al. (2018) confirmed that students with higher intrinsic learning motivation often obtain superior academic output. Clear learning objectives can effectively stimulate intrinsic learning motivation, making it easier for learners to perceive learning progress and gain a sense of achievement in the learning process.

As revealed in the study by Reeve and Cheon (2021), supportive learning contexts serve as a critical foundation for the effective implementation of instructional activities. The international students enrolled in preparatory courses generally exhibit homogeneous Chinese language proficiency, and the classroom teaching prioritizes the cultivation and practical application of language competence. Instructors can provide targeted guidance and personalized feedback in accordance with the individual learning needs of students, thus delivering highly personalized learning support. This type of supportive learning context can not only effectively improve the efficiency of Chinese language acquisition, but also significantly enhance the CLSE of international students.

Existing research on curriculum design indicates that, affected by the novice effect, beginner-level Chinese language courses for international students exhibit distinctive characteristics. This effect makes language learners more sensitive to linguistic input and more focused on their learning objectives. Beginner Chinese courses are characterized by: prioritizing survival-oriented communicative competence, adopting task-based modular teaching structures, arranging language knowledge and vocabulary repetition in a spiral progression, emphasizing timely instructional feedback, adopting context-driven multimodal input, and setting teaching content oriented toward cross-cultural adaptation. The overall curriculum framework is structured around the improvement of Chinese language proficiency, covering integrated courses, listening practice, speaking training and reading instruction. Despite the differences in course formats, unified teaching materials are generally adopted. While each course has a clear and differentiated teaching objective, they share the core goal of improving students’ language proficiency, consolidating basic knowledge, and enhancing cross-cultural adaptation ability. Through systematic curriculum arrangement, this design effectively improves international students’ CLSE, helping them smoothly pass the initial learning stage and form a positive learning cycle.

##### 4.2.1.5 Multilingual learning experience facilitates the transfer of new language learning strategies

This phenomenon can be explained by language learning experience, the application of learning strategies, cognitive advantages and cultural adaptability.

The cumulative experience of multilingual learning constitutes a critical factor in the enhancement of learning self-efficacy. International students with pre-existing multilingual learning backgrounds have a unique advantage in the process of Chinese learning: this accumulated experience enables them to adapt to the new language learning environment more quickly. For example, international students who have mastered other foreign languages have already developed mature learning methods for new languages, and are better able to predict and respond to various difficulties encountered in the process of language acquisition. This psychological preparation makes them more comfortable when engaging in Chinese learning, and further promotes the improvement of their self-efficacy. This conclusion is consistent with the research findings of Cheng and Bian (2023) on the self-efficacy of English learners.

The utilization of language learning strategies constitutes a core advantage of multilingual learners. The study by Cui and Hu (2022) confirms that the effective application of learning strategies can significantly improve learners’ self-efficacy in Chinese language acquisition. Multilingual international students generally master a richer system of language learning strategies, covering memory strategies, cognitive strategies, and metacognitive strategies. The adoption of diversified learning strategies can effectively improve learning efficiency and reduce learners’ academic frustration in the learning process. In addition, multilingual learners can flexibly transfer the successful learning experience accumulated in acquiring other languages to Chinese learning practice, which further promotes the improvement of their CLSE.

Enhancing cognitive flexibility and cultural adaptability is another key contributing factor. Students with multilingual backgrounds are generally exposed to diverse cultures, and thereby develop stronger cultural adaptability and more flexible cognitive thinking. This cross-cultural experience enables them to adapt to new environments more effectively and reduce psychological stress resulting from cultural differences. In Chinese language learning, students with high cultural adaptability and flexible cognition can often integrate into the Chinese-speaking environment more quickly, and leverage their linguistic advantages to further improve their language proficiency.

The sense of achievement among multilingual learners plays a vital role in their Chinese language acquisition. Students with prior language learning experience often make measurable progress in multilingual learning, and such successful experience significantly enhances their confidence in language acquisition. This confidence transfers to the process of Chinese learning, enabling learners to face learning challenges with stronger motivation, and ultimately improves their learning self-efficacy.

These factors make them have higher CLSE and can cope with the challenges in learning more easily.

##### 4.2.1.6 The high difficulty of learning Chinese creates a gap between international students’ expectations and reality

This phenomenon can be explained by multiple factors, including the rising difficulty of learning, the “mid-term fatigue” effect in language learning, the gap between expectations and reality, and changes in learning motivation.

The increasing difficulty of Chinese language learning is the primary contributing factor to the decline in learners’ self-efficacy. In the initial stage of Chinese learning, international students usually achieve rapid learning progress. At this stage, language acquisition mainly focuses on basic content, which is relatively accessible. Foundational knowledge such as core vocabulary, basic grammar and daily communication expressions can be mastered by learners relatively easily, which enables learners to obtain a strong sense of achievement and maintain a high level of learning self-efficacy. However, with the continuous advancement of learning, the inherent complexity of Chinese language gradually manifests, including nuanced differences in grammatical usage, the writing and memorization of Chinese characters, and the variations and application of lexical tones. These learning difficulties gradually become barriers to the continuous improvement of learners’ Chinese proficiency, which further increases the challenge of the Chinese learning process. When learners perceive a significant slowdown in their learning progress, their learning self-efficacy will naturally decline accordingly.

The “mid-term burnout” effect in language learning is another contributing factor to the decline in learners’ self-efficacy. The concept of learning burnout, which originates from the research on job burnout, was first proposed by American clinical psychologist Frodlandberg. He defines learning burnout as a phenomenon in which students develop negative attitudes towards academic studies: they lose enthusiasm for courses and campus activities, and experience obvious distraction in the learning process. Long-term continuous language learning often triggers learning burnout, and this is particularly prominent when learners enter the mid-stage of study. At this stage, learners may face problems including insufficient learning motivation and stagnant learning progress, which is referred to as the “mid-term burnout” effect. During this period, learners begin to question their own language learning ability, believing that they can no longer achieve obvious progress, which ultimately leads to a corresponding decline in self-efficacy.

The gap between learners’ expectations and their actual learning performance has a significant impact on learning self-efficacy. In the initial stage, international students usually hold high expectations for their own Chinese learning outcomes. After achieving rapid learning progress in the early period, they tend to maintain an optimistic expectation for subsequent learning results. However, with the advancement of learning, they gradually recognize the complexity of Chinese language and the protracted nature of the learning process, especially that it takes years of continuous learning to achieve advanced Chinese proficiency. When learners’ expectations conflict with the actual learning situation, students may experience obvious frustration, and believe that they cannot achieve the learning goals set at the initial stage. This cognitive dissonance undermines learners’ self-confidence, and ultimately leads to a decline in learning self-efficacy.

Changes in learning motivation are another key contributing factor. In the early stages of language learning, international students typically hold clear learning motivations, such as pursuing an academic degree or securing better job opportunities. However, as learning progresses, some students may gradually lose their initial learning drive, especially when they find that their goals are unattainable, or their progress falls short of their expectations. This waning motivation can give rise to self-doubt regarding their learning ability, and ultimately affects their learning self-efficacy.

Ultimately, the challenges of Chinese language learning become more prominent at the intermediate and advanced learning stages. As international students progress from the foundational learning stage to the intermediate stage, they are required to move beyond the mastery of basic communication skills and grasp complex academic and professional terminology. This not only increases the difficulty of learning but also amplifies learners’ psychological pressure. When facing such higher learning requirements, students are prone to suffer setbacks in learning confidence, as they cannot readily cope with the rising learning complexity.

#### 4.2.2 External reasons

##### 4.2.2.1 Insufficient social support leads to a decline in confidence

Social support theory holds that external support is critical to the development of learners’ learning confidence. Questionnaire results indicate that, on the dimension of “being accustomed to life in China” (item 43), the Chinese learning self-efficacy of non-ethnic Chinese international students is significantly lower than that of ethnic Chinese international students (*p =* 0.013). Furthermore, only 18 percent of all international students reported having fixed Chinese language learning partners (item 18). The lack of such support networks means non-ethnic Chinese students have to face dual challenges in cross-cultural adaptation: on one hand, the low level of family awareness of Chinese culture (34.7% of the surveyed students’ families do not understand China, item 17) weakens the emotional support that learners can obtain; on the other hand, limited social interaction opportunities (such as the lack of fixed language learning partners) make it difficult to transform classroom learning outcomes into practical communication skills. Especially for young learners aged 18 to 22, their anxiety in the dimension of affective attitude, such as the perception that “the progress of Chinese classes is too fast, and I worry about falling behind” (item 52, *M* = 3.07), partly originates from the absence of a buffer mechanism for extracurricular language practice. This uneven distribution of social support further exacerbates the self-efficacy gap among different groups of learners.

##### 4.2.2.2 The teaching content does not match the actual cultural needs

Research indicates that the high efficacy perception (*M* = 2.25) of preparatory students studying in China is correlated with the strong practical orientation of their curriculum. By contrast, as undergraduate and postgraduate students advance in their studies, their academic courses become disconnected from practical needs in daily life. For example, the widespread anxiety identified in the affective attitudes dimension regarding “difficulty writing Chinese characters” (item 59, *M* = 3.07) and “concern about insufficient Chinese proficiency” (item 50, *M* = 3.11) reflects the insufficient decoding ability of advanced learners for cultural symbols. Additionally, among non-Chinese ethnic Chinese international students, some come from family backgrounds where family members “can understand Chinese but cannot speak it” (25.1%, item 16). This background may lead them to regard Chinese merely as a communication tool rather than a cultural carrier, which further weakens their learning motivation. This mismatch between personal needs and academic content causes some long-term learners to fall into a vicious cycle of decreasing CLSE, and ultimately leads to the absence of effective competency verification.

##### 4.2.2.3 The ripple effect of cross-cultural anxiety

Conscious emotional contagion refers to the phenomenon in which an individual consciously recognizes the emotional states of other individuals or groups, identifies the underlying causes of others’ emotions, and then generates corresponding shared emotions by placing themselves in others’ positions (Falkenberg et al., 2008).

Low levels of familiarity with Chinese culture among international students’ family members (item 17) may trigger feelings of isolation for international students, and this pattern is particularly prominent among non-ethnic Chinese international students. This subgroup shows significantly lower self-efficacy scores on item 43, which measures respondents’ adaptation to life in China. Within the affective attitude dimension, two factors—“feeling tense in Chinese language classes” (item 38, *M* = 3.07) and “anxiety about learning progress” (item 52, *M* = 3.07)—further aggravate the negative emotions of international students, and exert adverse impacts on the overall learning atmosphere. In the absence of effective emotional support mechanisms, such as professional psychological counseling services or structured peer support systems, this anxiety can spread from individual students to the entire student group, ultimately leading to a further decline in collective Chinese learning self-efficacy.

### 4.3 Recommendations for improving the CLSE of international students

#### 4.3.1 Enhancing learners’ sense of language objectives

Research indicates that preparatory students exhibit the highest efficacy sense (*M* = 2.20) due to the “novice effect,” whereas undergraduates (*M* = 2.61) and postgraduates (*M* = 3.36) show declining efficacy as learning progresses (*p* = 0.005). To address this, establishing clear language learning objectives for international students is crucial, as goal orientation serves as the core driver of learning motivation. Chen et al. (2015) highlight that insufficient learning motivation is a prevalent issue in regular higher education institutions, and learners without clear goal orientation often feel disoriented and struggle to maintain sustained engagement.

Educators should therefore help students develop personalized learning plans by decomposing distant objectives into short-term, actionable tasks. This phased approach not only allows students to track their progress but also boosts their confidence, thereby enhancing learning self-efficacy. Regular goal reviews and assessments provide students with periodic achievements, helping them avoid self-doubt. In addition, learners should also learn to set specific goals. Teachers can guide students to develop goal-setting skills and encourage them to record their learning progress. This not only improves learning efficiency but also helps them persevere when facing difficulties.

#### 4.3.2 Developing learners’ self-regulation abilities

In order to solve the problem of weak internal motivation of young learners, we can develop learners’ self-regulation ability in various ways to improve their CLSE. Self-regulation is a key factor in the success of international students in learning Chinese. It involves the ability to plan, monitor, adjust, and evaluate their learning process. Bouffard-Bouchard et al. (1991) found that self-regulation and self-efficacy are closely linked, with students who demonstrate strong self-regulation skills often achieving better academic performance and higher self-efficacy.

In practical teaching activities, educators can apply metacognitive strategies to support students in improving their self-regulation capabilities. Schools can organize special workshops to guide students in self-management of academic tasks and optimization of learning strategies. In addition, through the mutual assistance mechanism established by study groups, students can improve their proficiency in applying learning strategies through communication and discussion, thereby improving their overall learning self-efficacy.

#### 4.3.3 Reduce the impact of anxiety and improve the positive emotional experience of learners

The affective attitudes dimension had the lowest mean value of learning self-efficacy (2.81) among the four dimensions, with particularly prominent issues of classroom tension (item 38, *M* = 3.07) and Chinese character writing anxiety (item 59, *M* = 3.07). Anxiety is a key factor affecting learning efficacy. Many international students experience heightened anxiety when learning Chinese characters and complex grammatical structures, as evidenced in this study’s assessment of learning self-efficacy. This emotional state not only hampers academic performance but also undermines their confidence, ultimately diminishing their motivation to learn.

To reduce learners’ anxiety, teachers can construct an inclusive and reassuring classroom learning environment, encouraging students to make bold attempts while minimizing the negative impacts of learning errors. Through targeted emotional support, teachers can help international students alleviate academic pressure, and thereby improve their CLSE. Additionally, schools can provide standardized psychological counseling and support services to offer assistance to students who experience anxiety caused by academic pressure.

The cultivation of positive learning emotions is also of equal significance. Educational institutions can strengthen students’ learning interest by implementing a variety of diversified teaching activities, including cultural exchange initiatives and language practice courses. In the course of participating in these activities, students can not only put their Chinese language proficiency into practical application, but also engage in interaction with peers and instructors in a relaxed environment, which in turn enhances both their learning motivation and self-confidence.

#### 4.3.4 Developing learners’ ability to use learning strategies

Learning strategies exert a direct impact on students’ academic performance. Cui and Hu (2022) found that a number of international students lack effective learning strategies, particularly in the selection of learning methods and time management. Oliver and Purdie (1999) also demonstrated that higher frequency of learning strategy application is correlated with greater learning self-efficacy, which indicates a bidirectional relationship between these two factors.

Schools and teachers can support students in improving their ability to apply learning strategies by providing targeted and specific guidance. For example, educators can introduce practical classroom strategies including mind mapping, keyword memorization, and phased review methods, which effectively assist students in retaining and mastering Chinese knowledge. Furthermore, schools can offer specialized learning strategy training programs to help students develop personalized learning approaches and apply them flexibly in practical learning contexts.

Furthermore, the development of learners’ learning strategies can be promoted through practical learning engagement. Schools can provide students with more hands-on learning opportunities, such as Chinese language corners and speech contests, to enable learners to apply Chinese knowledge in authentic language contexts. This approach not only consolidates the knowledge students have acquired, but also enhances their confidence in their own learning abilities, and thus improves their learning self-efficacy.

#### 4.3.5 Enhance cultural adaptation support and establish a diversified social support system

Non-Chinese students presented lower self-efficacy in the social support dimension (*M* = 2.53), which is mainly attributable to two factors: 40% of these students’ family members lack Chinese language proficiency, and the students themselves have limited opportunities for social interaction (only 18% of them have language partners). International students studying in China face cultural adaptation challenges, which is an important external factor affecting their self-efficacy in Chinese language learning. Language and cultural differences leave them feeling unfamiliar and isolated when they first arrive in China, which reduces their quality of life and weakens their confidence in Chinese learning. Therefore, schools and society should strengthen support for cultural adaptation to help students overcome relevant barriers, establish sound social relationships, and improve their learning efficacy. Schools can help international students adapt to Chinese culture by organizing cross-cultural exchange activities. Encouraging interaction between Chinese and international students enables the latter to adapt to Chinese social norms and cultural environments through communication and cultural exchange.

The social support system is crucial for enhancing international students’ CLSE. Schools should strengthen interactions between international students, teachers and peers, and create more opportunities for Chinese language practice. By organizing study groups, language partner programs, and cultural exchange activities for international and Chinese students, schools can encourage international students to use Chinese more frequently, and boost their confidence in language proficiency through practical application. Empirical evidence shows that more frequent social interactions and more authentic language use scenarios correspond to higher learning self-efficacy among international students. Schools may collaborate with local communities and cultural institutions to organize social activities such as cultural experience programs, volunteer services and festival celebrations. This allows international students to perceive social support in a broader social context, and such diversified social support helps them adapt to China’s cultural and social environment more quickly, and enhances their learning confidence and self-efficacy through practical language use.

## 5 Conclusion

This study employed a questionnaire-based approach to examine the current state of Chinese Language Learning Self-Efficacy (CLSE) among 427 international students, and the collected data were processed and analyzed using SPSS statistical software. The findings indicate that the overall CLSE level of the participating international students falls within the moderately high range. For analytical purposes, this study divides learning self-efficacy into four dimensions: cognitive ability, affective attitude, learning strategy, and social support. Among these dimensions, the affective attitude dimension yielded the lowest self-efficacy score (*M* = 2.81), which demonstrates that the surveyed international students generally lack adequate confidence in their Chinese language learning capabilities. This cohort reported significant anxiety across multiple areas, including the accuracy of assignment completion, performance in Chinese language courses, final learning outcomes, and Chinese character reading and writing, with all relevant mean scores exceeding 3.0. Regarding the social support dimension, international students’ Chinese learning self-efficacy was higher than that observed in the other three dimensions (*M* = 2.53). For the two sub-components “family support for Chinese language learning” and “support beneficial for future planning,” international students’ Chinese learning self-efficacy was at a moderately high level, with mean scores (of 2.27 and 2.38, respectively).

Female international students exhibit a marginally higher overall level of self-efficacy in Chinese language learning compared to their male counterparts. In the cognitive ability dimension, participants aged 23 to 27 years demonstrate greater confidence. Ethnic Chinese international students display stronger self-efficacy in both the affective attitude dimension and the social support dimension. Across participants at different educational stages, college preparatory students achieve the highest self-efficacy scores, a phenomenon attributable to the novice effect. Multilingual students show more distinct advantages in the learning strategy dimension. Among international students with over 3 years of Chinese language learning experience, self-efficacy decreases alongside the rising difficulty of Chinese language acquisition.

Limited by research constraints, the present study analyzed a sample of just over 400 international students. Although the survey outcomes can partially reflect the current state and existing issues of international students’ CLSE, the sample is restricted to a single higher education institution located in southeastern coastal China, due to the limited timeframe allocated for questionnaire data collection. As a result, the conclusions drawn in this study cannot fully generalize to the overall population of international students regarding their CLSE. For subsequent research, it is recommended that the survey scope be expanded to incorporate international student cohorts from diverse institutions and geographic regions, enabling the acquisition of more multi-dimensional research data.

Against the backdrop of globalization, the population of language learners studying Chinese as a second language has exhibited sustained growth. Improving Chinese as a Second Language Self-Efficacy (CLSE) among international students can effectively facilitate their ultimate Chinese learning outcomes. Subsequent scholarship may broaden the scope of investigation to conduct further empirical inquiry into the current situation of CLSE among international students studying in China, and explore how to strengthen international students’ CLSE through language teaching interventions, learning strategy guidance, and social support. This approach will enable them to address challenges encountered in Chinese learning more effectively and ultimately improve their Chinese learning outcomes.

## Data Availability

The original contributions presented in the study are included in the article/supplementary material, further inquiries can be directed to the corresponding author.
